# Adaptive and Phase Selective Spike Timing Dependent Plasticity in Synaptically Coupled Neuronal Oscillators

**DOI:** 10.1371/journal.pone.0030411

**Published:** 2012-03-06

**Authors:** Victor Kazantsev, Ivan Tyukin

**Affiliations:** 1 Dept of Nonlinear Dynamics, Institute of Applied Physics of RAS, Nizhny Novgorod, Russia; 2 Dept of Neurodynamics and Neurobiology, University of Nizhny Novgorod, Nizhny Novgorod, Russia; 3 Dept of Mathematics, University of Leicester, Leicester, United Kingdom; University of Zaragoza, Spain

## Abstract

We consider and analyze the influence of spike-timing dependent plasticity (STDP) on homeostatic states in synaptically coupled neuronal oscillators. In contrast to conventional models of STDP in which spike-timing affects weights of synaptic connections, we consider a model of STDP in which the time lags between pre- and/or post-synaptic spikes change internal state of pre- and/or post-synaptic neurons respectively. The analysis reveals that STDP processes of this type, modeled by a single ordinary differential equation, may ensure efficient, yet coarse, phase-locking of spikes in the system to a given reference phase. Precision of the phase locking, i.e. the amplitude of relative phase deviations from the reference, depends on the values of natural frequencies of oscillators and, additionally, on parameters of the STDP law. These deviations can be optimized by appropriate tuning of gains (i.e. sensitivity to spike-timing mismatches) of the STDP mechanism. However, as we demonstrate, such deviations can not be made arbitrarily small neither by mere tuning of STDP gains nor by adjusting synaptic weights. Thus if accurate phase-locking in the system is required then an additional tuning mechanism is generally needed. We found that adding a very simple adaptation dynamics in the form of slow fluctuations of the base line in the STDP mechanism enables accurate phase tuning in the system with arbitrary high precision. Adaptation operating at a slow time scale may be associated with extracellular matter such as matrix and glia. Thus the findings may suggest a possible role of the latter in regulating synaptic transmission in neuronal circuits.

## Introduction

Spike timing dependent plasticity (STDP) is one of the simplest yet key mechanisms enabling functional adaptation in neuronal systems (see e.g. [1] and references therein). Broadly speaking, if we consider two synaptically connected cells, STDP stands for a change in synaptic efficacy as a function of timing between pre- and post- synaptic events. If the pos-synaptic event occurs within a given interval of time from the onset of the pre-synaptic one then efficacy of synaptic transmission enhances. If, however, the opposite takes place, i.e. a post-synaptic event is followed by pre-synaptic spike, then the efficacy decreases. Despite overall apparent simplicity of the phenomenon, it allows to link higher cognitive functions such as learning and memory with molecular and cellular processes underlying signal transmission in neuronal networks. Various interesting aspects of STDP in relation to bidirectional plasticity and bistability have been discussed and analyzed in the literature [2]–[4]. In addition, as it has been shown in [5], STDP may be involved in the formation of metaplasticity [6]. With respect to the function, STDP is a component of plausible models of selective attention [7] and working memory [8]. At the lower scale of functional organization, STDP may trigger long-term potentiation (LTP) or depression (LTP) [9]–[12]. Finally, STDP is believed to play a role in phase coding – a way of representing information about stimuli in terms of the relative time moments of spike occurrences.

Many forms of STDP have been discovered to date [13], and a common knowledge is that STDP is supported by multiple molecular cascades inducing changes in both postsynaptic spines and in presynaptic terminals. Calcium flux through NMDA receptors located in spines [14] is an example of mechanisms directly responsible for postsynaptic changes. In this mechanism, excitatory postsynaptic potentials preceding back-propagating action potentials elicit calcium influx through postsynaptic NMDA receptors. Higher calcium concentration, in turn, facilitates evoking of postsynaptic spikes in response to the presynaptic ones. Changes in presynaptic terminals are observed, for example, in the hippocampal mossy fiber synapses [15]. STDP-like phenomena can also occur due to the modulation of synaptic transmission by endocannabinoid-mediated retrograde cascades. These cascades, once activated, trigger the activation of presynaptic receptors [16], [17].

Large diversity of the ways in which STDP may manifest itself in empirical observations has lead to a broad range of mathematical models of the phenomenon. These models, although phenomenological, are widely used in computational and theoretical studies (see e.g. [18]–[21]). In the majority of these models the principal factor determining synaptic efficacy is the synaptic weight. The latter is described by a dynamic variable of which the value changes in response to post-to-presynaptic spike timing. Increments/decrements of the weights are often associated to LTP/LTD respectively. One of the outcomes of such activity-dependent modifications of the synaptic weights is that connections between individual cells may grow or decay over time by a relatively large amount. This facilitates emergence of neuronal clusters that fire together, up to a tolerance margin.

A particular form of such firing activity in which clusters of neurons produce time-locked *spiking sequences* has recently received substantial attention in the literature [22]–[25]. Relative time lags between spikes in these sequences are robust; the sequences can repeat spontaneously, or they can be generated in response to a certain stimulus. A number of theoretical frameworks have been proposed to explain emergence and persistence of these precise firing patterns with different inter-spike timing, see e.g. [23] and related notions of synchronized chains (synfire chains) and polychronous groups. In these frameworks STDP, linked to the post-to-presynaptic timing, is advocated as a mechanism that is directly responsible for the emergence of persistent spike sequences within a given topological substrate. Even though computational evidence suggests that this may indeed be the case, rigorous correspondence between stimuli, particular STDP-based signaling pathways, and their stability is not yet fully understood. In particular, the question of how STDP may ensure precise timing of spiking sequences with arbitrary lags between spikes is still open. Finding an answer to this question is the main goal of our current work.

In this paper we investigate dynamic properties of a pair of neural oscillators coupled via synaptic STDP-enabled connections. Our results suggest that for this class of systems accurate tuning of post-to-presynaptic spike timing to a given, and broadly arbitrary, value is indeed possible via a suitable STDP mechanism. This mechanism can be viewed as a feedback facilitating or depressing synaptic transmission “on demand”, depending on timing of stimulation. In contrast to conventional models of STDP in which spike-timing modulates weights of synaptic connections, we consider a model of STDP in which spike-timing influences internal state of pre- or post- synaptic neurons. Such internal state is, in the case of our model, an excitation parameter enhancing/suppressing spike generation. This feature of spike-dependent potentiation is well-documented phenomenologically [26]. We show that coarse tuning of spike timing is readily achievable in a pair of interconnected neural oscillators equipped with such STDP mechanism. Further fine-tuning of spiking patterns can be achieved via additional slow fluctuations of the base line of excitation thresholds.

The main motivation for choosing excitation-driven STDP mechanisms rather than conventional models of STDP (i.e. the ones modulating the weights of connections) is that we would like to be able to deal with realistic cases of neurons having different natural frequencies. As a general rule, the larger the difference between natural frequencies of neural oscillations the larger should be the values of synaptic weights if accurate time-locking of spikes is desired, cf. e.g. [21], [27]. This, however, may conflict with the standard assumption demanding that coupling between elements in the system is weak. Thus regulatory mechanisms complementary to the ones modulating the values of synaptic weights are needed for ensuring precise locking of spike sequences in systems of neurons with inherently non-identical frequencies of spike generation. STDP-driven modification of excitation variables is a plausible candidate for this role.

For the sake of numerical and analytical tractability we focus predominantly on a simplified spike transmission model using a pair of neuronal oscillators coupled via excitatory synaptic coupling. Synaptic transmission in the model is unidirectional and instantaneous: a spike in the postsynaptic neuron is evoked as soon as the excitatory postsynaptic potential (EPSP) exceeds certain threshold. As a model for pre- and post-synaptic neurons we use Rowat-Selverston neuronal oscillator [28]. This model is computationally efficient, yet being a reduction of Hodgkin-Huxley classical model, it bears a fair degree of biological realism. The model is typically used in computational studies of synchronization and phase-locking in networks of synaptically coupled cells [29]. Here we also employ this model for studying phase-locking behavior of neurons with STDP-enabled synaptic connections.

The paper is organized as follows. Section *Methods* contains description of the Rowat-Selverston neuronal oscillator and also specifies the class of synaptic coupling considered in the paper. In addition, it presents the concept of phase spiking maps which is used in both numerical and analytical parts of the study. Definitions of specific STDP mechanisms are provided in *Results*. This is followed by quantitative and qualitative description of the dynamics such mechanisms may induce in the coupled system. The results are summarized and discussed in a brief *Discussion*. Technical derivations and other auxiliary materials are presented in *Appendix S1*.

## Methods

### Synaptically coupled neuronal oscillators

We studied dynamical properties of a pair of spiking neuronal oscillators coupled by an excitatory synapse [28], [29]. Each neuronal oscillator in this system is a computationally efficient reduction of standard Hodgking-Huxley equations; oscillators of this type have been used widely in computational neuroscience in the context of synchronization [29]. Since we did not intend to focus on any specific molecular mechanisms of synaptic transmission but rather were concerned with mere dynamics of spikes, picking this model in favor of other alternatives strikes a plausible balance between biological realism and overall computational efficiency. Mathematically, the model can be expressed as follows:
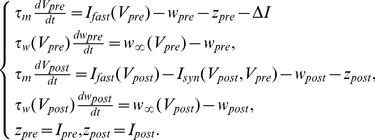
(1)Subscripts 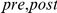 in (1) label variables governing dynamics of presynaptic and postsynaptic neurons, respectively. Variables 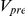, 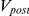 stand for the corresponding membrane potentials. Parameters 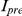, 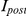 model constant currents determining equilibrium depolarization levels; 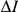 is the difference in depolarization (hence, natural spiking frequencies) between two neurons. The function
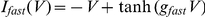
models fast currents across cell membrane, and 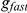 is the conductance of the fast voltage-dependent inward current, Variables 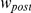, 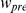 are the slow recovery variables, and 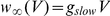 is the voltage-dependent activation function; 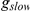 is the corresponding conductance.

Time scales of the spikes are determined by parameter 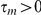 and the function
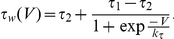
The function 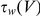 is the voltage dependent characteristic time of the slow current, and 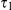, 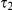, 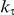 are parameters. We consider the case when 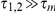 and 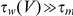, and 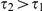. This ensures that duration of individual spikes is small relative to the inter-spike intervals.

Synaptic current in (1) is implemented in accordance with the following instantaneous synaptic transmission model:
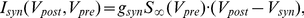
(2)where 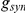 is the maximal synaptic conductance reflecting synaptic strength. Function
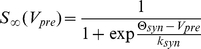
(3)defines the amount of available neurotransmitter, and parameters 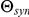 and 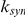 characterize the midpoint and slope of synaptic activation, respectively. Parameter 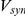 is associated with the synaptic reversal potential; it controls the sign of synaptic currents induced by spikes at the presynaptic neuron. In this model, the synapse is excitatory if 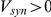. Hence, because we consider the case when the neurons are connected by an excitatory synapse, we set 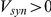. The values of all relevant parameters of the model are provided in Table 1.

**Table 1:** Parameters of model (1).

Parameter	Values
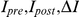	0.5, 0.5, [−1.1 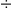 0.2]
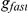 , 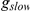	2.0, 2.0
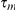 , 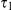 , 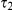 , 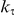	0.16, 5.0, 50.0, 0.05
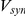 , 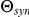 , 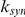 , 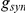	1.0, 0.0, 0.16, [0.0 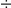 1.0]

**Figure 1:**
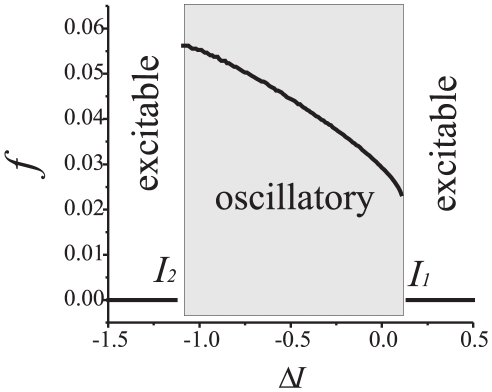
Spike oscillation frequency (e.g. natural frequency) as a function of the level of depolarization in single neuron model described by **Eqs. (1)**. The values of frequency 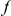 are computed for the dimensionless model.

When 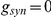 pre- and post-synaptic oscillators are uncoupled, both producing sequences of pulses with constant, albeit different, firing rate. Periodic oscillations in each uncoupled compartment appear through the supercritical Andronov-Hopf bifurcation [30], [31]. In terms of Eqs. (1), such bifurcation occurs when parameter 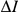 reaches some critical value. This mimics depolarization of the membrane by a constant current injection. Dependence of the spiking rates on the depolarization levels is illustrated in Fig. 1. In Fig. 1, labels 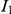 and 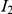 mark maximal and minimal values of 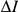 for which the dynamics of both compartments is oscillatory. Note that, in principle, there are very narrow intervals to the left of 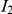 and to the right of of 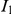 in which low-amplitude oscillations exist. These are not shown in the figure. If the values of 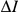 are outside of a small neighborhood of this interval then the system is in the excitable mode. If 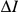 is within the interval 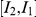 then the frequency curve, 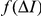, is a strictly monotone and continuous function. Thus in this interval there is a one to one correspondence between the depolarization parameter 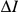 and the spike firing rate, 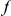.

### Spiking phase map

In order to characterize and analyze post- to presynaptic timing in (1), including cases when 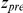 and 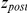 are varying with time, we introduce *spiking phase map* [32]. The map itself is constructed as follows. First, we define the relative spiking phase, 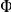, as:
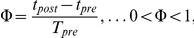
where 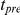 is the time corresponding to occurrence of a presynaptic spike, and 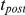 is the time of the first postsynaptic spike generated in response to the presynaptic one; 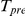 is the period of oscillations in the presynaptic neuron. Variable 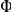, therefore, may be viewed as a sample of relative phase of the oscillators that is measured at the moments of time when the post-synaptic oscillator fires. Second, having defined a sequence of 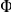 over time, we determine the *spiking phase map* as follows:
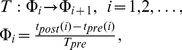
(4)where 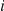 is the index of transmitted spikes in the sequence.

It was shown in [32] that in the case of constant 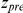, 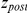 transformation (4) may be modeled by a one-dimensional point map, 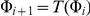, where 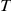 is a piece-wise continuous function on the interval 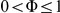. Stable fixed points of this map correspond to the spike synchronization mode 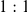. Spiking phase in this mode is locked to the value of the fixed point. Note, that the *spiking phase map* can be also viewed as a discrete version of the *pulse coupled equations*. These are typically used in the literature on the analysis of weakly coupled neuronal oscillators for describing dynamics of relative phases in the system. The function 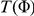 in this context is often referred to as the *phase response curve* (PRC). The advantage of using discrete spiking phase map instead of its continuous-time counterpart is that the discrete map, (4), is defined for any values of coupling strengths, provided that both systems oscillate.

**Figure 2:**
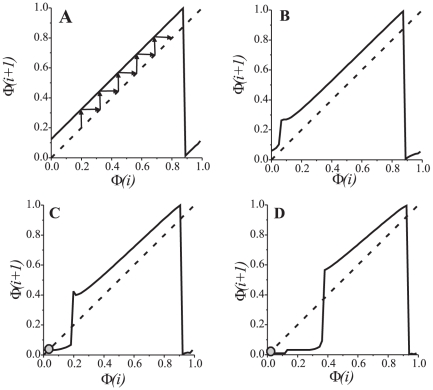
PRC curves for different coupling strengths,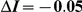. A: 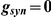. Relative phase shift is monotonically increasing as it is shown by arrows. The increase is linearly proportional to the frequency mismatch, 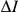. B: PRC for small value of the synaptic coupling, 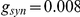. Monotonically increasing phase is pulled towards the abscissa in the vicinity of the origin. C: Synchronization for 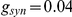. Stable fixed point emerging from the tangent (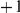) bifurcation defines the value of the phase locked with a small synaptic transmission delay. D: Synchronization for the increased coupling strength, 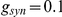. The fixed point is close to zero.

**Figure 3:**
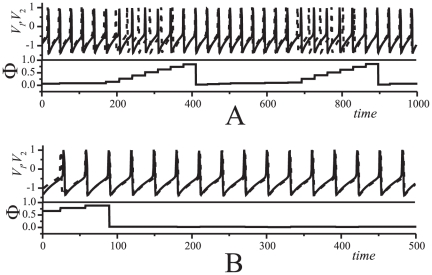
Oscillations in synaptically coupled oscillators **Eqs. (1)**. Upper panel shows the membrane potentials in presynaptic (dashed curve) and in postsynaptic neurons (solid curve), respectively. The lower panel shows time evolution of the relative spiking phase. A: Phase pulling effect. Long lasting quasi-synchronous signals are alternating with phase reset intervals. Parameter values: 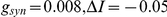. B: Synchronization and phase locking due to the excitatory synaptic coupling. Parameter values: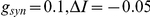.

Figure 2 shows typical shapes of the PRCs for (1). In the absence of coupling relative phase shifts increase in a monotone fashion (Fig. 2A). Adding a small coupling alternates the dynamics and, respectively, PRCs. Figure 2 B shows the spiking phase map near the tangent or 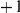 bifurcation. There appears to be a region (a ghost) in the figure which is pulling and trapping, for quite a long period of time, the values of 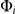. The effect is illustrated in more detail in Fig. 3A. Notice that the system's state may remain in a neighborhood of the synchronous mode for a rather long time. In the phase space of Eqs. (1) this corresponds to solutions near periodic or quasi-periodic orbits on the invariant torus. Further increase of 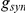 leads to appearance of a stable fixed point. The fixed point corresponds to nearly synchronous firing (i.e. with almost zero phase lags) of pre and post- synaptic oscillators (Fig. 2C,D). An example of such a solution of (1) is shown in Fig. 3B.

**Figure 4:**
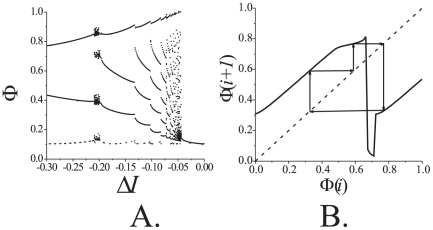
Dynamics of spiking phase map (4) for oscillators with different natural frequencies. A: Bifurcation diagram illustrating dependence of the relative spiking phase on frequency mismatch. Parameter values: 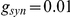. B: Example of periodic trajectory of the spiking phase map which corresponds to 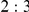 spike frequency ratio.

Dependence of the spiking phase map for (1), and hence the dynamics of (1), on other parameters of the system is illustrated with the one-parameter bifurcation diagrams provided in Fig. 4. Fig. 4A shows the values of 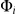 when the coupling strength, 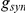, is fixed but parameter 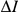 is varying. In agreement with standard intuition, the presence of sufficiently strong synaptic coupling results in nearly synchronous oscillations if the natural frequencies mismatch, 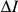, is relatively small. When the value of 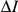 increases synchronous 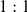 mode disappears. Instead of the synchronous mode stable periodic trajectories emerge (Fig. 4B). These correspond to periodic motions on a torus in the phase space of (1). According to the figure (Fig. 4A), periodic modes with different rotation numbers may be followed by intervals of complex (quasiperiodic or chaotic) dynamics.

## Results

### Model of STDP

**Figure 5:**
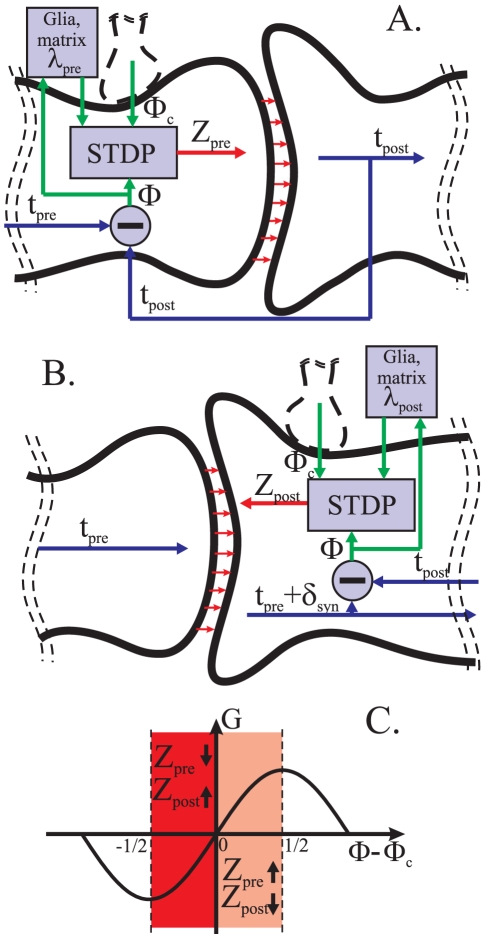
Schematic representation of the *adaptive* STDP phase locking. Timing between the postsynaptic and the presynaptic spikes is modeled by the spiking phase, 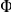. The difference between 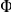 and 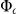, where 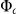 is some reference value that might be induced by another regulatory inputs, activates the feedback mechanism, “STDP”; the latter activates molecular cascades changing the state, denoted by 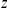, of the presynapse and/or postsynapse. Direct STDP feedback is modulated by fluctuations of extracellular medium, 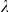 (e.g. the metaplasticity), giving rise to the adaptation, i.e. fine tuning of the phase-locked state. A: Presynaptic STDP feedback. B: Postsynaptic STDP feedback. C: STDP curves used in simulations. Positive half-period of the 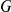-function indicates potentiation by the increase of presynaptic frequency and/or depression by the decrease of postsynaptic frequency.

We propose a phenomenological model of synaptic transmission in a pair of spiking neuronal oscillators supplied with an *adaptive* STDP regulatory mechanism. A diagram describing this mechanism is schematically presented in Fig. 5. The diagram shows two possible ways in which the timing of spikes may influence state of synaptic coupling.

*The first alternative* is illustrated in Fig. 5A. Timing of pre- and post-synaptic spikes is affecting the state of the presynaptic neuron. Such change of the neuron's state is accounted for in the model by a phenomenological variable 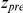. Increasing/decreasing the value of 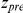 facilitates/depresses transmission of stimuli, respectively. Such spike-timing-modulated signal transmission in the model acts as a feedback relating timing of pre-to-post synaptic spikes with the neuron's excitability parameter 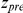.

Dynamics of this phenomenological variable, 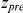, is driven by an STDP function curve of which the shape depends on specific molecular mechanisms. Here, for illustrative and computational purposes, we model this curve by a simple function resembling a truncated sinusoid (Fig. 5C). This STDP curve determines dependence of 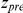 on relative time differences between post- and presynaptic spikes (e.g. relative spiking phase). These relative time differences are denoted by 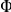 (see Methods).

In addition to the relative spiking phase, 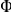, the model accounts for an optional phase offset, 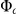. The latter can be added to or subtracted from the value 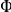. The origins of this extra variable are many: it can account e.g. for the influence of delays inherent to signal transmission in neural circuits; it may also model external inputs to the presynaptic neuron. In the context of our present work we will view variable 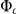 as a *reference* relative phase: the relative phase between spikes which is to be attained asymptotically. In addition to the STDP curve and the phase offset 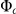, we also introduce a regulatory parameter 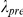. This extra parameter determines the baseline to which the values of 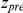 relax in absence of stimulation. In the model it accounts for small and relatively slow fluctuations of extracellular medium. One can speculate that these fluctuations could be related to glia and matrix influence on synapses - the subject which has been discussed in many empirical studies [33]. The latter fluctuations affect the function of STDP and thus they can also be related to metaplasticity [6].

*The second alternative* is illustrated in Fig. 5B. Here spike-timing affects the state of the postsynaptic neuron. Spikes arriving to terminals of the presynaptic neuron cause the release of a neurotransmitter. The neurotransmitter reaches the postsynaptic neuron, and this triggers generation of postsynaptic potentiation (PSP) with latency time 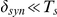 (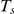 is the characteristic time scale of the spike train, e.g. the period of oscillations). In this model PSP, in turn, triggers generation of the response spike (e.g. action potential). The latter event is then detected in the postsynaptic terminal via a chemically or electrically back-propagating signal. Similarly to the previous (presynaptic) case there is a state variable 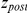 whose increase or decrease facilitates potentiation or depression, respectively. Other parameters of this mechanism such as 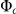 and 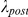 are similar to the case discussed in the first alternative.

Let us now formulate the STDP models discussed above mathematically. Consider a pair of spiking neuronal oscillators coupled by an excitatory synapse (see Equations (1) in Methods) [28], [29]. The original equations are extended according to the circuitry shown in Fig. 5. Presynaptic STDP feedback (shown in Fig. 5 A) is governed by the following equations:
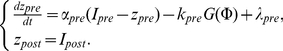
(5)Similarly, postsynaptic STDP has the form:
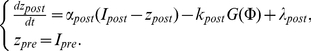
(6)In essense, Eqs. (5) and (6) are additional currents in the presynaptic and postsynaptic neurons, respectively. The current are dependent on spike-timing. Parameters 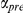, 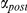 stand for the time scales of the polarization's relaxation, and 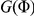 accounts for the STDP curve. Parameters 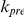, 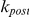 are gains. Function 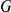 is in the right-hand side of (5), (6) is assumed to be bounded, sufficiently smooth, and “1”-periodic. In particular, the following is supposed to hold:
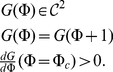
(7)Variable 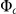 in (7) is the reference phase, 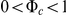. In the present work, for simplicity, we select the function 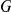 as follows:
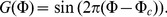
(8)


In the next section we analyze dynamics of the combined system (1), (5) and (6) when the values of 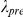, 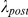 are fixed, and natural frequencies of pre- and post-synaptic oscillators are not identical.

### STDP with presynaptic feedback

**Figure 6:**
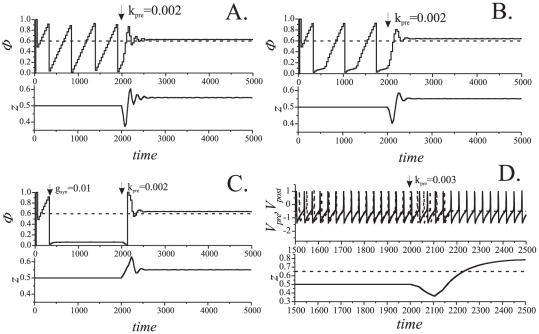
Dynamics of two neuronal oscillators with presynaptic control. Upper and the lower panels show the evolution of the relative phase shift (in A–C) and control variable, 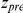, respectively. A: No synaptic coupling. Parameter values: 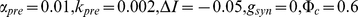. B: Phase pulling mode. Parameter values: 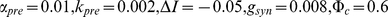. C: Switching the phase locking mode from the unsupervised mode (defined by the synaptic coupling) to the one enslaved by the reference phase. Parameter values: 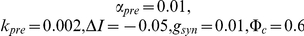. D: Failure of the phase control due to overregulation effect. The upper panel shows membrane potentials in two neurons. Parameter values: 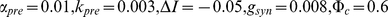.

Consider system (1), (5), and (8). Dynamics of this configuration for 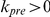 is illustrated in Figure 6A. One can observe that, after a relatively short transient behavior, the relative phase, 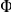, locks near the reference value, 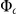. According to the figure, the transient looks like damped oscillation relaxing asymptotically to a stable fixed point. When the relative phase locks presynaptic neuron changes its depolarization level (Fig. 6A–C, lower panel). Notice that locking occurs for both zero and nonzero synaptic coupling. Figure 6B illustrates dynamics of the system in the phase pulling mode (see Methods, Fig. 2B). If the coupling between cells is made relatively strong then presynaptic STDP feedback may destroy the in-phase synchronization mode and switch the system into the phase-locked mode determined by the value of reference phase (Fig. 6C).

The values at which relative phase locks are determined by the values of the control variable, 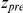, at the fixed point. The values of 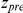 and relative phase at the fixed point (denoted by 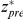 and 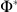 respectively) can be determined from (5):
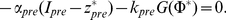
(9)Hence, according to (8) the value of phase locking mismatch, 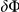, can be estimated as follows
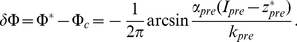
(10)The larger is the value of 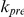, the higher is the precision of phase locking. Notice, however, that if the feedback gain, 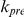, exceeds a critical threshold, the STDP phase locking regulatory mechanism described above may fail. Loss of stability of the fixed point is a possible explanation for this observation. For extremely large values of 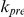 one can observe an “overregulation” catastrophe (Figure 6D). In short, STDP suppresses presynaptic neuron so hard that the neuron is eventually driven into excitable mode. This is shown in the upper panel of Fig. 6D. The value of 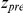 exceeds the critical value, 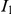 (see Methods), and the presynaptic neuron becomes inhibited: no spikes are evoked.

**Figure 7:**
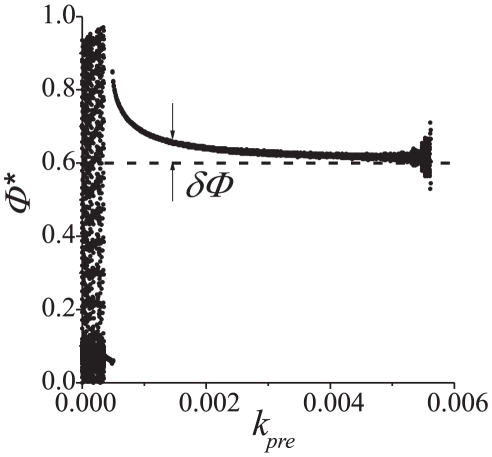
Phase control bifurcation diagram. Values of the outcome phase 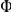 driven by Eqs. (5) versus feedback strength, 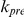. Parameter values: 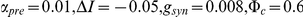.

In order see the range of parameters for which presynaptic STDP can be considered as a viable phase locking mechanism we calculated numerically dependence of 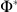 on 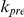 (Fig. 7). When 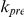 is small the relative phase 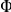 is not settling to a particular constant value; it “scans” through the whole interval of admissible values, 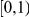. If 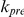 is increased beyond a threshold value the relative phase locks. Increasing the value of 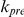 further results in locking of relative phase in a neighborhood of the reference, 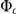, as predicted by (10).

With regards to the influence of STDP model (5) on behavior of the coupled system an interesting phenomenon can be observed: in-phase oscillations become apparently stable at some critical value of 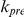 (lower left corner of the plot). In other words, presynaptic STDP facilitates existing synaptic connections by providing synaptic efficacy equivalent to stronger synaptic coupling (transition from Fig. 2B to Fig. 2C in Methods). For larger values of 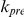 relative phase 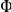 jumps to a neighborhood of the reference phase 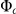. According to the figure, increments of 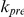 (in a relatively broad interval) result in improvements of the phase locking accuracy: relative phase 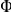 approaches 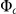 with the growth of 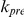. There is, however, a critical value of 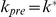 at which the fixed point becomes neutrally stable. Further increments of 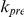 result in destabilization of the fixed point.

In order to assess stability of the relative phase dynamics we invoke the idea of spiking phase maps (see Methods). Here the one-dimensional spiking phase map discussed in Methods is extended as follows:
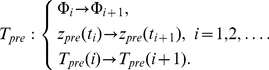
(11)Variable 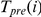 is the period of presynaptic spikes; it is now time-varying due to the STDP feedback. Since there is a functional dependence between 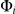 and 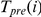, map (11) can be approximated by a two-dimensional one describing dynamics of the variables 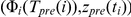.

**Figure 8:**
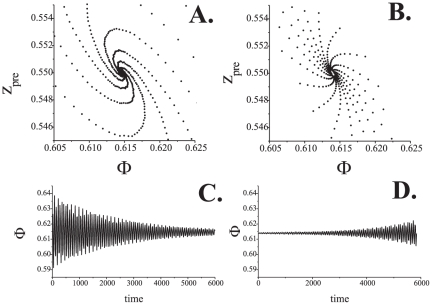
The dynamics of map (11) in the vicinity of the Neimark-Saccer bifurcation point,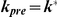. A and C. Phase plane dynamics and the oscillation profile near stable fixed point for 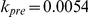. B and D. Phase plane dynamics and the oscillation profile near unstable fixed point for 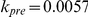. Parameter values: 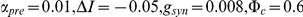.

Investigating dynamics of (1), (5), (8) numerically we have found that the critical gain 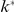 corresponds to the neutral stability of 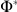 with zero real part of its complex conjugate multipliers. Therefore, Neimark-Saccer bifurcation takes place at 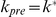 [30]. Figure 8 shows trajectories of the spiking phase map in the vicinity of 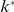. One can see from this figure that if 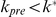 then variables 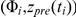 travel towards the stable fixed point (see Figs. 8 A and C). If, however, 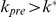 then 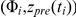 move in the opposite direction (see Figs. 8 B and D), and the fixed point appears to be unstable. This behavior indicates that the bifurcation is subcritical (with positive first Lyapunov coefficient). Thus, for 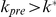 relative phase 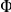 oscillates with a growing amplitude (Fig. 8 D). One can also observe that for 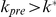, which are some distance apart from 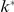, variable 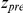 (after a short transient) leaves the domain corresponding to the oscillatory mode (Fig. 1 in Methods). This, in turn suppresses all oscillations in the presynaptic neuron.

In the bifurcation diagram in Fig. 7 a “cloud” of points emerges when 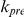 approaches the critical point 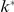 from the left. The size of this cloud grows with 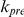 in a seemingly continuous way. This contrasts with our earlier remark about that the bifurcation is subcritical. Notice, however, that if 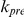 approaches 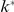 from the left, real parts of the linearized map's eigenvalues are becoming negligibly small, and also the convergence rate to the fixed point is asymptotically decreasing to zero. Since numerical simulations were run over given and finite interval of time, the amplitude of this cloud, i.e. deviations of 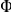 from the fixed point at the end of the simulation, depends explicitly on the convergence rate of the map. The smaller is the convergence rate the higher are the chances that deviations of 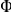 from 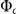 are larger at the end of the simulation. This is exactly what we observe in the figure.

### STDP with postsynaptic feedback

Consider the second mechanism of the postsynaptic STDP feedback – the one in which timing of pre- and post- synaptic events changes excitability of the postsynaptic neuron (Fig. 5A). In this case dynamics of the presynaptic neuron is not affected. Hence it is plausible to assume that the presynaptic neuron generates a sequence of spikes with a fixed, albeit unknown, frequency.

Lest us investigate dynamics of relative phase for this system. As before, we approach the task by constructing and analyzing the corresponding phase spiking map (see (4), Methods). Given that the value of 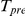 is constant, the map is described as follows:
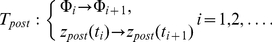
(12)Yet, for the sake of convenience of illustration we will only present its one-dimensional projections on the relative phase coordinate, 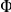.

**Figure 9:**
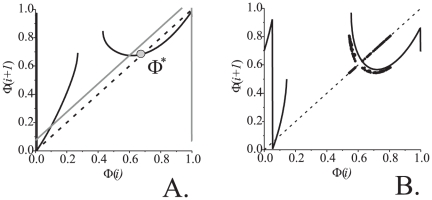
The PRCs for the one-dimensional approximation of the spiking phase map (12). A: Appearance of the stable fixed point for 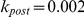 indicating phase locking mode in the signal transmission with reference phase 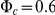. Grey curve shows the PRC without control. B: The PRC for large control strength, 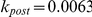, indicating the appearance of chaotic attractor. The dots show the trajectory of the two-dimensional map (12). Parameter values: 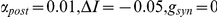.

**Figure 10:**
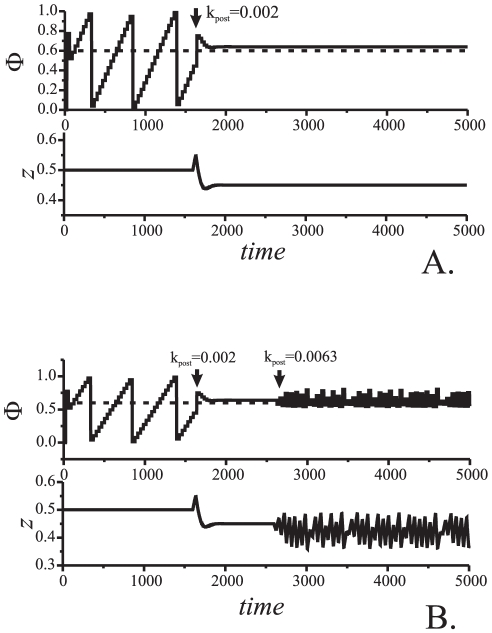
Evolution of spiking phase and control variable 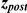 for postsynaptic control. A: Phase locking. Parameter values: 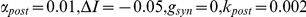. B: Chaotic oscillation of the spiking phase near the reference phase. The strength of the feedback is changed in two steps marked by the arrows. Parameter values: 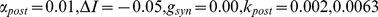.

Similarly to what has been observed for the first alternative, STDP feedback stabilizes relative phase in a neighborhood of the reference value. Corresponding PRCs are shown in Fig. 9A. The figure suggests presence of a stable fixed point, 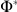. If one increases the value of 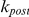 the fixed point 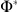 looses stability through the period doubling bifurcation. To the right of this critical point behavior of the system resembles a route to chaos through the period doubling cascade (Fig. 9B) [30]. In contrast to the previously considered configuration (presynaptic STDP feedback), in this case relative phase remains in a vicinity of the fixed point even if the fixed point itself becomes unstable. The values of relative phase, however, appear to be attracting to a stable 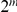-periodic orbit or to a set with a structure of a chaotic attractor. Corresponding plots of the evolution of 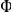 and 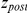 are shown in Fig. 10A,B. Further increments of 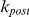 lead to a catastrophe of the attractor. The catastrophe occurs because the values of 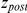 become so large that oscillations in the postsynaptic neuron disappear (see Fig. 1, Methods).

The fact that a set on which the values of 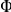 project resembles an object looking strikingly similar to a chaotic attractor suggests a rather unexpected function of the STDP mechanism considered here. The function is that such STDP-induced dynamics may offer a natural facility for encoding of information in the system. Indeed, if this set is a chaotic attractor then it comprises of infinite number of orbits with varying periods. Thus, in principle, a rich set of spiking sequences can be activated in such a system if an appropriate stimulus arrives.

**Figure 11:**
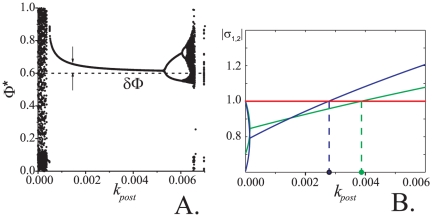
Bifurcation and stability diagrams for the case of postsynaptic control. *Left panel:* phase control bifurcation diagram. Values of the outcome phase 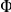 driven by Eqs. (6) versus feedback strength, 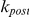. Parameter values: 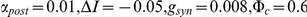. *Right panel:* stability diagram derived from the local analysis of the fixed points of (S1.5) in Appendix S1. Blue line shows the values of 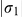, 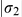 (eigenvalues of the Jacobian of (S1.5), see also (S1.9)) as functions of 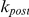 for 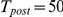. Green line depicts the values of 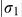, 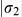 for 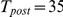. Other parameter values were set as follows: 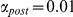, 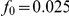, 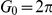, 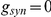. Blue and green circles indicate critical values of 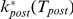, for 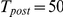 and 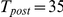 respectively, at which the fixed point 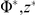 becomes unstable. Notice that stability diagram in the right panel (derived analytically) is largely consistent with the bifurcation diagram in the left panel (obtained by means of numerical simulations). Slight inconsistencies are evident in the area where 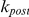 are small. These inconsistencies are due to that 1) our analytical derivations ignore the influence of synaptic coupling, 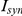, and that 2) the fixed point may disappear when 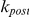 small.

Bifurcation diagrams characterizing dynamics of the system are shown in Fig. 10. When the values of 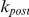 are relatively small the picture is similar to the case of presynaptically-driven feedback (Fig. 7). If we increase the value of 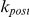 (up to the first critical point), relative phase will eventually lock to a value corresponding to nearly in-phase oscillations. Again, the phenomenon is very similar to the case of presynaptic configuration: STDP facilitates in-phase oscillations even if the synaptic connection is relatively week. If 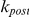 is increased even further (until the second critical value) relative phase locks near the reference 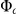. Further increments of 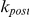 result in gradual improvements of accuracy until, however, 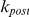 arrives at the third critical value. At this point the period doubling bifurcation occurs in the spiking phase map (12). Increasing the value of 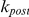 beyond this critical point gives rise to the bifurcation cascade. The latter, in turn, leads to emergence of chaotic-looking dynamics [30], [31] of the relative phase (Fig. 9B, Fig. 10B). This state, however, is also limited in terms of the range of admissible values of 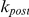. If 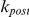 becomes too large, i.e. it exceeds the forth critical value, oscillations in the postsynaptic neuron disappear (Fig. 11A).

**Figure 12:**
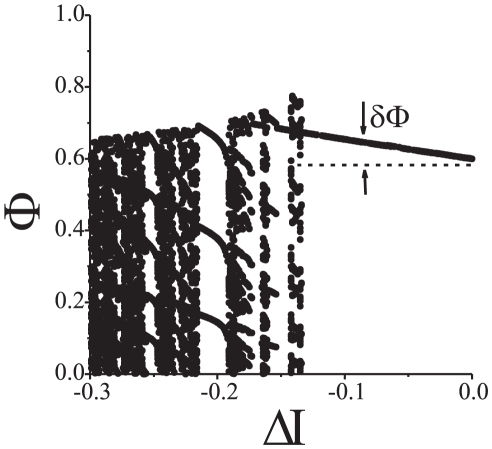
Spiking phase locking modes for increasing natural frequency mismatch. Illustration for the case of presynaptic control (5) Parameter values: 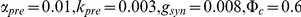.

In addition to numerical simulations we analyzed stability of the fixed point analytically. The results are presented in Appendix S1 and also are illustrated with stability diagrams in Fig. 10. We have shown that when the system is in the phase locking mode the fixed point is exponentially stable. Hence, the dynamics persists under small perturbations. A somewhat more detailed, albeit complicated, picture emerges from numerical simulations. In particular, Figure 12 illustrates how fluctuations of the depolarization level, 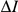, may affect dynamics of phase locking for a fixed value of 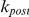. As expected, there is a frequency band in which spiking phase remains locked. Phase locking error 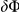 grows if the frequency mismatch, 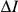, increases in absolute value. When the values of 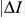 become relatively large synchronous mode disappears, and different periodic, quasiperiodic and chaotic motions emerge. Qualitatively, this resembles the case of direct synaptic coupling (see Methods, Fig. 4). Similar scenarios were observed in the system with postsynaptic feedback (6).

### Adaptive phase-locking STDP

So far we considered two spike-timing regulatory mechanisms ensuring stable phase locking in the system. According to these results, both mechanisms guarantee locking of relative phases of oscillations a vicinity of the reference subject to the choice of parameters. Yet, as one can see from these results too, phase locking occurs with an error. Dynamics of the system in a neighborhood of the phase locking state, e.g. for the case of postsynaptic feedback, satisfies the following inequality (see (S1.12), Appendix S1)
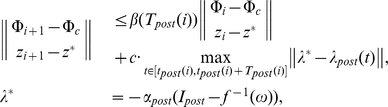
where 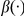 is a decreasing strictly monotone function such that 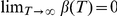, 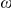 is the frequency de-tuning parameter (see (S1.1)–(S1.3) in Appendix S1), 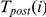 is the interval between spikes at 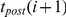 and 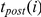, 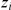 stands for 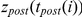, 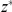 is the value of 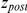 at the equilibrium when 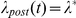, 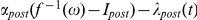 is the term characterizing the amplitude of the relative phase fluctuations around desired values at 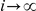, and 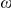 is the natural frequencies mismatch.

According to this, (see also (10) and (S1.11) in Appendix S1) if parameters of the STDP law are chosen such that 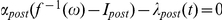 then the relative phase variable, 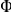, (in a neighborhood of the locking state) locks to the the reference 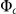 asymptotically. The problem is, however that the value of natural frequencies mismatch, 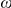, is unknown a-priori. Thus annihilating the error by choosing the values of 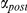, 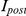 (or 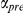, 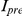 for the presynaptic feedback) is not a viable option. On the other hand, the possibility for minimizing the error by assigning large values to 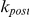 (or 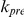) is also limited. This is because, as we have shown analytically (see (S1.7), (S1.9) in Appendix S1) and demonstrated numerically (Fig. 9B), increasing the values of 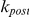 leads inevitably to the loss of attractivity of the fixed point.

Nevertheless, as we illustrate below, asymptotic reduction of the phase-locking error to zero can be achieved via adjustments of 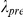 or 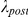 according to a simple adaptation mechanism. This adaptation mechanism is in essence a slow fluctuation of the excitation thresholds. The frequency of these fluctuations increases if absolute values of relative phase are far away from the desired ones. The frequency slows down when relative phase approaches its desired value, i.e. the reference 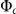.

The most simplest model of such fluctuations is, perhaps, the following:
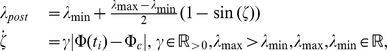
According to [34], [35] (see also Appendix S1, (S1. 16) and Proposition 1) such adaptation scheme ensures that 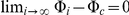 provided that the value of 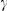 is sufficiently small and 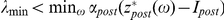, 
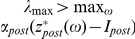
. A very similar adaptation mechanism can be derived for 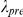 as well by replacing subscripts 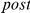 with 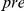 in the above. Dynamics of adaptive phase-locking STDP in (1) with variable 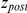 evolving according to (6) is illustrated in Fig. 12. According to the figure, when extracellular adaptation feedback is activated the error of phase locking is slowly vanishing with time.

## Discussion

In the previous sections of the manuscript we demonstrated how an STDP mechanism affecting neuronal excitability can be used for tuning of time lags between presynaptic and postsynaptic spikes. Even though the model we studied is obviously a simplification the resulting regulatory mechanisms may still be considered as biologically plausible (see e.g. Fig. 5 illustrating timing dependent modulations of state of presynapse, postsynapse and extracellular matter). Numerical and analytical studies of the model revealed that the values of time lags between pre- and post- synaptic events can be maintained with remarkably high accuracy. In fact, if no external perturbations are present then the accuracy can be made arbitrarily high. Thus the study demonstrates that STDP mechanisms linked to neuronal excitability can play an important role in explaining key characteristics, such as e.g. pre- post- synaptic timing, of signal transmission in the brain.

Precise timing of signals in the system can be achieved via assigning appropriate values to internal parameters of the STDP mechanism. These are the reference phase, 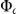, strength/slope of the STDP's action, 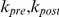, time constants 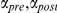, and excitation baseline parameters 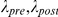.

The mechanism itself can be viewed as a feedback steering relative phase of the spikes towards a desired reference value. As opposed to more simplistic modeling views in which synapses are treated as mere physical connections with only one regulatory parameter, the synaptic gain, our study shows that dynamics of synapses and synaptic connections constitute a significant addition. So much so that systems equipped with such dynamic connections become capable of adapting to inherent differences of prior excitation in the cells. In addition they may also compensate for the discrepancy of natural frequencies in the connected neurons. This creates an analysis framework for generating and testing existence of dynamic functional architectures not only in a pair of non-identical neurons but also in networks of cells. Thanks to explicit connection between parameters of STDP and values to which relative phase converges, we hope that similar connections may potentially be established at the level of networks too.

**Figure 13:**
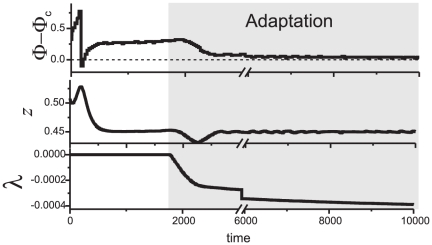
Adaptive compensation of phase locking errors via extracellular adaptation feedback. Illustration for the case of presynaptic control (5). Parameter values: 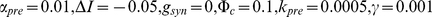.

In addition to demonstrating potential of STDP with regards to regulating spike timing to a vicinity of some desired reference value we investigated the problem further. In particular, we studied a possibility of making spike timing in the system arbitrarily accurate. We demonstrated that introduction of a simple STDP adaptation circuit enables to achieve highly accurate tuning of spike timing in the system for a wide range of values of the reference phase (Figs. 5, 13 illustrate location of this circuit in the mechanism and show how the system with such circuit may function). Adaptation here refers to a process of self-tuning of internal parameters of the synapse in response to deviations of spike timings from the desired ones. As we have shown in previous sections, if natural frequencies of oscillations are not identical then spike timing in systems with non-adapting STDP circuits is likely to deviate from the reference. The error can not be eliminated by making the values of gains of STDP large. This is because such an increase will inevitably lead to instabilities. We showed, however, that a synapse with adaptation in just one parameter of STDP, namely 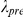 or 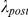, maintains desired spike timing with arbitrarily high precision. The process can be thought of as slow fluctuations of “state of extracellular matter”. At the present level of biophysical detail used in our simulations we could not associate such process explicitly with a specific extracellular molecular cascade. Nevertheless, we can speculate that certain characterizations of the 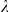 processes (e.g. low strength influence, relatively slow time scale, integration effect) are quite similar to the influence of glia and extracellular matrix on synaptic transmission described in [33].

Concluding, we summarize key outcomes of our study are as follows:

- We propose a robust computational solution for task-oriented STDP; the mechanism is capable of stabilizing given post-to-presynaptic spike timing with arbitrary high precision.
- Both presynaptic and postsynaptic STDP feedbacks regulating internal neuronal excitation enable stable maintenance of the desired spike timing values.
- The task-oriented STDP needs additional adaptation feedback, possible mediated by extracellular matter e.g. glia and matrix, if precise spike-timing or low gains in the presynaptic and postsynaptic feedbacks are required.
- Higher gains in STDP postsynaptic feedback may trigger complex modes of phase dynamics with periodically or chaotically fluctuating post-to-presynaptic spike timing values.

## Supporting Information

Appendix S1 **Supplementary material including additional analytical results on stabilizing effect of STDP and phase adaptation.**
(PDF)
