# Supplementary material for: Adaptive and Phase Selective Spike Timing Dependent Plasticity in Synaptically Coupled Neuronal Oscillators
Source: PLoS One. 2012 Mar 6;7(3):e30411. doi: 10.1371/journal.pone.0030411 (PMC3295799; doi:10.1371/journal.pone.0030411)
Supplement: Appendix S1 — Supplementary material including additional analytical results on stabilizing effect of STDP and phase adaptation. (PDF) [file pone.0030411.s001.pdf]

Appendix S1. Supplementary materials for

Adaptive and phase selective spike timing dependent plasticity in synaptically coupled neuronal oscillators

Victor Kazantsev[1,2,*] Ivan Tyukin[3]
1 Dept of Nonlinear Dynamics, Institute of Applied Physics of RAS, Nizhny Novgorod, Russia
2 Dept of Neurodynamics and Neurobiology, University of Nizhny Novgorod, Nizhny Novgorod, Russia
3 Dept of Mathematics, University of Leicester, Leicester, United Kingdom
∗ E-mail: vkazan@neuron.appl.sci-nnov.ru

Relative phase dynamics and stabilizing effect of STDP

For the sake of simplicity we consider the case when only one of the two coupled phase oscillators, e.g. postsynaptic, is supplied with the STDP-type mechanism. We will also assume that if the value of z_{post} is fixed (within an interval) then both oscillators operate in a vicinity of asymptotically stable limit cycles. In addition, we will suppose that the values of g_{syn} are negligibly small so that we can investigate stabilizing effects of STDP in the combined system independently from effect of phase pulling due to the instantaneous synaptic coupling between oscillators [1].

Under the assumptions above, phase of the oscillations in V_{pre}, w_{pre}-system (see e.g. [2]) can be defined as a function $\phi_{pre}(V_{pre}, w_{pre}, \Delta I)$ such that

$$\frac{\partial \phi_{pre}}{\partial V_{pre}} \dot{V}_{pre} + \frac{\partial \phi_{pre}}{\partial w_{pre}} \dot{w}_{pre} = \omega_0(I_{pre} + \Delta I), \tag{S1.1}$$

where $\omega_0(I_{pre} + \Delta I)$ is the natural frequency of oscillations. Since the frequency of oscillations decreases with ΔI (see Fig. 13), the function $\omega_0(\cdot)$ is strictly monotone and non-increasing. Moreover, for a technical reason we will assume that the function $\omega_0(\cdot)$ is differentiable or that it can be approximated by a differentiable function in the domain of interest.

Similarly, if the value of z_{post} is fixed, phase of the oscillations in V_{post}, w_{post}-system can be expressed as a function $\phi_{post}(V_{post}, w_{post}, z_{post})$ such that

$$\frac{\partial \phi_{post}}{\partial V_{post}} \dot{V}_{post} + \frac{\partial \phi_{post}}{\partial w_{post}} \dot{w}_{post} = \omega_0(z_{post}), \tag{S1.2}$$

Notice that if the value of z_{post} is allowed to vary and $|\dot{z}_{post}|$ is sufficiently small, then $\dot{\phi}_{post} \simeq \omega_0(z_{post})$ provided that $|\omega_0(z_{post})| \gg |\dot{z}_{post}|$. The latter inequality reflects that the frequency of oscillations is much higher than the time constant of the STDP. In what follows we will consider the case when this asymptotic holds.

Taking (S1.1), (S1.2) into account we can conclude that dynamics of the relative phase, φ, is to satisfy the following equation

$$\dot{\varphi} = \omega_0(z_{post}) - \omega_0(I_{pre} + \Delta I).$$

Denoting $\omega_0(z_{post}) - \omega_0(I_{pre} + \Delta I) = \omega - f(z_{post})$, where $f(\cdot)$ is a continuous, locally Lipschitz, strictly monotone non-decreasing function, and $\omega \in \mathbb{R}$ is the frequency de-tuning parameter, we arrive at the following system of equations:

$$\begin{aligned} \dot{\varphi} &= \omega - f(z_{post}) \\ \dot{z}_{post} &= \alpha_{post}(I_{post} - z_{post}) + k_{post} G(\sigma(\varphi, t, i) - \Phi_c)) + \lambda_{post}, \end{aligned} \tag{S1.3}$$

where

$$\sigma(\varphi, t, i) = \Phi_i, \quad t \in [t_{post}(i), t_{post}(i) + T_{post}(i)).$$

With regards to the values of ω, we will assume that $\omega \in \text{range}(f)$. It is also clear that $\varphi(t_{pre}(i)) = \Phi_i$, $i = 1, 2, \dots$ according to the definition of variable Φ_i in (4).

Let $t \in [t_{post}(i), t_{post}(i) + T_{post}(i))$, then the last equation in (S1.3) can be explicitly integrated giving rise to

$$\begin{aligned} z_{post}(t) =& e^{-\alpha_{post}(t - t_{post}(i))} z(t_{post}(i)) + \frac{k_{post}}{\alpha_{post}}(1 - e^{-\alpha_{post}(t - t_{post}(i))}) G(\sigma(\varphi, t, i) - \Phi_c)) \\ &+ \left(\frac{\lambda_{post}}{\alpha_{post}} + I_{post}\right)(1 - e^{-\alpha_{post}(t - t_{post}(i))}) \end{aligned} \tag{S1.4}$$

Thus taking (S1.3) and (S1.4) into account and approximating the function $f(\cdot)$ by a linear one, $f(z_{post}) = f^* + f_0 z_{post}$, we can now express $\varphi(t)$ for $t \in [t_{post}(i), t_{post}(i) + T_{post}(i))$ as:

$$\begin{aligned} \varphi(t) =& \varphi(t_{post}(i)) + \omega(t - t_{post}(i)) - f^*(t - t_{post}(i)) - f_0\Big[\frac{1}{\alpha_{post}}(1 - e^{-\alpha_{post}(t - t_{post}(i))}) z(t_{post}(i)) \\ &+ \left(\frac{k_{post}}{\alpha_{post}} G(\sigma(\varphi, t, i) - \Phi_c) + \left(\frac{\lambda_{post}}{\alpha_{post}} + I_{post}\right)\right)\left((t - t_{post}(i)) - \frac{1 - e^{-\alpha_{post}(t - t_{post}(i))}}{\alpha_{post}}\right)\Big]. \end{aligned}$$

Noticing that $\varphi(t_{post}(i) + T_{post}(i)) = \Phi_{i+1}$, $\varphi(t_{post}(i)) = \Phi_i$, denoting $z_i = z_{post}(t_{post}(i))$, $z_{i+1} = z_{post}(t_{post}(i) + T_{post}(i))$, and using the fact that $\varphi(t)$ is continuous in t, we arrive at

$$\begin{aligned} \Phi_{i+1} =& \Phi_i + \omega T_{post}(i) - f^* T_{post}(i) - f_0\Big[\frac{1}{\alpha_{post}}(1 - e^{-\alpha_{post} T_{post}(i)}) z_i \\ &+ \left(\frac{k_{post}}{\alpha_{post}} G(\Phi_i - \Phi_c) + \left(\frac{\lambda_{post}}{\alpha_{post}} + I_{post}\right)\right)\left(T_{post}(i) - \frac{1 - e^{-\alpha_{post} T_{post}(i)}}{\alpha_{post}}\right)\Big] \\ z_{i+1} =& e^{-\alpha_{post} T_{post}(i)} z_i + \left(\frac{k_{post}}{\alpha_{post}} G(\Phi_i - \Phi_c) + \left(\frac{\lambda_{post}}{\alpha_{post}} + I_{post}\right)\right)(1 - e^{-\alpha_{post} T_{post}(i)}) \end{aligned} \tag{S1.5}$$

Let Φ^*, z^* be an equilibrium of (S1.5). Given that $G(\cdot)$ is differentiable, and neglecting dependence of $T_{post}(i)$ on z_{post} if z_{post} is close enough to z^* , we can linearize the dynamics of (S1.5) about Φ^*, z^*:

$$\begin{pmatrix} \Phi_{i+1} - \Phi^* \\ z_{i+1} - z^* \end{pmatrix} = K_i \begin{pmatrix} \Phi_i - \Phi^* \\ z_i - z^* \end{pmatrix}, \; K_i = \begin{pmatrix} k_{11}(i) & k_{12}(i) \\ k_{21}(i) & k_{22}(i) \end{pmatrix} \tag{S1.6}$$

where

$$\begin{aligned} k_{11}(i) =& 1 - \frac{f_0 G_0 k_{post}}{\alpha_{post}}\left(T_{post}(i) - \frac{1 - e^{-\alpha_{post} T_{post}(i)}}{\alpha_{post}}\right) \\ k_{12}(i) =& -\frac{f_0}{\alpha_{post}}(1 - e^{-\alpha_{post} T_{post}(i)}) \\ k_{21}(i) =& \frac{G_0 k_{post}}{\alpha_{post}}(1 - e^{-\alpha_{post} T_{post}(i)}) \\ k_{22}(i) =& e^{-\alpha_{post} T_{post}(i)} \\ G_0 =& G'(\Phi^*, z^*). \end{aligned} \tag{S1.7}$$

Denoting $\delta\Phi = \Phi^* - \Phi_c$ (cf. (10)) we rewrite (S1.6) as

$$\begin{pmatrix} \Phi_{i+1} - \Phi_c \\ z_{i+1} - z^* \end{pmatrix} = K_i \begin{pmatrix} \Phi_i - \Phi_c \\ z_i - z^* \end{pmatrix} - u_i, \; u_i = \begin{pmatrix} k_{11}(i) - 1 \\ k_{21}(i) \end{pmatrix} \delta\Phi. \tag{S1.8}$$

where, according to the second equation in (S1.5), the value of $\delta\Phi$ is

$$\delta\Phi = \frac{1}{G_0}\left(\frac{-\lambda_{post} - \alpha_{post}(I_{post} - z^*)}{k_{post}}\right).$$

Let σ_1, σ_2 be eigenvalues of the matrix

$$K_i = \begin{pmatrix} k_{11}(i) & k_{12}(i) \\ k_{21}(i) & k_{22}(i) \end{pmatrix}$$

Then, provided that the fluctuations of $T_{post}(i)$ are sufficiently small, condition $|\sigma_1| < 1,\ |\sigma_2| < 1$ ensures that the fixed point Φ^*, z^* is stable in the sense of Lyapunov.

The eigenvalues of K_i can be expressed as

$$\sigma_{1,2} = \frac{(k_{11}(i) + k_{22}(i)) \pm \sqrt{(k_{11}(i) + k_{22}(i))^2 - 4(k_{11}(i)k_{22}(i) - k_{12}(i)k_{21}(i))}}{2}. \tag{S1.9}$$

According to (S1.7), (S1.9), checking if $|\sigma_1| < 1,\ |\sigma_2| < 1$ holds requires availability of the estimates/values of f_0 and G_0. The value of f_0 can be explicitly inferred from Fig. 1: $f_0 \simeq 0.025$, and the value of G_0 belongs to $[0, 2\pi]$. Figure 11 (right panel) shows plots of $|\sigma_1|$, $|\sigma_2|$ as functions of k_{post} for $\alpha_{post} = 0.01$, $G_0 = 2\pi$, and two different values of T_{post}: $T_{post} = 35$ (green curve), and $T_{post} = 50$ (blue curve). According to the figure, there is a range of values of k_{post} for which both $|\sigma_1|$ and $|\sigma_2|$ are less than one, and hence the fixed point is stable. The boundaries of this range are largely consistent with numerical analysis of the original system summarized in the bifurcation diagram in Fig. 11 (left panel). Minor disagreements between the figures can be observed for k_{post} small. These, however, are due to the following two factors. First, the fixed point itself disappears when the values of k_{post} become sufficiently small. Second, model (S1.3) does not include the influence of synaptic coupling, I_{syn}. Neither of these factors are accounted for in expression (S1.9), and that is why the stability diagram in Fig. 11 (right panel) is inconsistent with the bifurcation one (left panel) for k_{post} small.

Summarizing the analysis above one can conclude that

- if $f_0, G_0 > 0$ then, for a broad range of T_{post}, there will always exist values of the STDP parameters, k_{post}, α_{post}, such that the fixed point of (S1.5) is locally exponentially stable for these values;
- if the values of k_{post} are made large enough, i.e. when $\max\{|\sigma_1|, |\sigma_2|\} > 1$ (which is always possible to achieve, see (S1.7), (S1.9)), the corresponding fixed point becomes unstable.
- on the other hand, if the values of k_{post} are too small then the fixed point ceases to exist.

An alternative strategy for assessing stability of the equilibria of (S1.3) can be carried out without explicit integration of the second equation in (S1.3). If $k_{post} \in \mathbb{R}_{>0}$, $\alpha_{post} \in \mathbb{R}_{>0}$ are sufficiently small then solutions of (S1.3) can be approximated by that of

$$\begin{aligned} \dot{\varphi} &= \omega - f(z_{post}) \\ \dot{z}_{post} &= \alpha_{post}(I_{post} - z_{post}) + k_{post}G(\varphi - \Phi_c)) + \lambda_{post}. \end{aligned} \tag{S1.10}$$

It is clear that equilibria of (S1.10) can be determined from

$$\begin{aligned} z^* &= f^{-1}(\omega) \\ \Phi^* &\in G^{-1}\left(\frac{-\alpha_{post}(I_{post} - f^{-1}(\omega)) - \lambda_{post}}{k_{post}}\right) + \Phi_c \end{aligned} \tag{S1.11}$$

where G^{-1} is, in general, a set-valued function. If Φ^* is such that $\partial G/\partial\varphi(\Phi^* - \Phi_c) > 0$ then one can conclude that Φ^*, z^* is an asymptotically stable equilibrium of (S1.3). The conclusion follows from the analysis of the time-derivative of the following Lyapunov candidate:

$$V(\varphi, z_{post}) = \int_{z^*}^{z_{post}} (f(s) - f(z^*))ds + k_{post} \int_{\Phi^*}^{\varphi} G(s - \Phi_c) - G(\Phi^* - \Phi_c)ds$$

followed by invoking the Barbalatt's lemma for demonstrating asymptotic convergence of $\varphi(t)$ to Φ^*.

An important consequence of the stability analysis above, specifically (S1.3) – (S1.8), is that if λ_{post} is allowed to vary then, subject to the choice of k_{post}, α_{post}, the dynamics of (1) in which z_{post} evolves in accordance with (6) locally satisfies the following constraint:

$$\begin{aligned} &\left\| \begin{array}{c} \varphi(t) - \Phi_c \\ z_{post}(t) - z^* \end{array} \right\| \leq \beta(t - t_0) \left\| \begin{array}{c} \varphi(t_0) - \Phi_c \\ z_{post}(t_0) - z^* \end{array} \right\| + c \cdot \max_{t \in [t_0, t]} \|\lambda^* - \lambda_{post}(t)\|, \ t \geq t_0 \\ &\lambda^* = -\alpha_{post}(I_{post} - f^{-1}(\omega)), \end{aligned} \tag{S1.12}$$

where $\beta(\cdot)$ is a strictly monotone, positive, and non-increasing function vanishing asymptotically at infinity, and c is a non-negative constant.

Phase adaptation

As before we will suppose that only one oscillator (postsynaptic) is equipped with the STDP mechanism, i.e. only λ_{post} is adapting. Consider system (S1.3) in which the function $\lambda_{post}(t)$ evolves according to the following simple rule:

$$\lambda_{post}(t_0) + \Gamma \int_{t_0}^{t} |\sigma(\varphi, \tau, i) - \Phi_c| dt \leq \lambda_{post}(t) \leq \lambda_{post}(t_0) + \gamma \int_{t_0}^{t} |\sigma(\varphi, \tau, i) - \Phi_c| dt, \ \Gamma, \gamma \in \mathbb{R}_{>0}, \tag{S1.13}$$

where $\sigma(\varphi, t, i) = \Phi_i$ for all $t \in [t_{post}(i), t_{post}(i) + T_{post}(i))$, $T_{post}(i) > 0$, and $|\lambda^* - \lambda_{post}(t_0)| \leq M_\lambda$. The value of M_λ is supposed to be small enough so that there is a neighborhood of Φ_c: $|\varphi - \Phi_c| \leq M_\Phi$ such that (S1.12) holds for all

$$|\varphi(t) - \Phi_c| \leq M_\Phi, \ |\lambda^* - \lambda_{post}(t)| \leq M_\lambda. \tag{S1.14}$$

According to Proposition 1 (see next section), if at any given t_0 variables $\varphi(t_0), \lambda_{post}(t_0)$ of the combined system (S1.12), (S1.13) satisfy

$$|\varphi(t_0) - \Phi_c| \leq M_\Phi, \ 0 \leq \lambda^* - \lambda_{post}(t_0) \leq M_\lambda$$

then picking

$$0 \leq \gamma \leq \beta^{-1} \left(\frac{d}{\kappa} \right)^{-1} \frac{\kappa - 1}{\kappa} \frac{M_\lambda}{\beta(0)(M_\Phi + c|u|(1 + \kappa/(1 - d))) + cM_\lambda} \tag{S1.15}$$

ensures that $\varphi(t)$ converges to Φ_c asymptotically.

Consider a modified version of (S1.13):

$$\lambda_{post}(t) = \lambda_{\min} + \frac{\lambda_{\max} - \lambda_{\min}}{2} \left(1 - \sin \left(\frac{2}{\lambda_{\max} - \lambda_{\min}} \left(\lambda_0 + \gamma \int_{t_0}^{t} |\sigma(\varphi, \tau, i) - \Phi_c| d\tau \right) \right) \right), \tag{S1.16}$$

where the value of γ is chosen according to (S1.15), and $\lambda^* \in [\lambda_{\min}, \lambda_{\max}]$. Suppose that for any continuous $\lambda_{post}(t)$: $\lambda_{\min} \leq \lambda_{post}(t) \leq \lambda_{\max}$

1) solutions of (S1.3) are defined;

2) for each λ_{post} fixed, $\lambda_{post} \in [\lambda_{\min}, \lambda_{\max}]$, there is a unique attractor, which is locally exponentially stable;

3) there is a neighborhood of Φ_c : $|\varphi - \Phi_c| < M_\Phi$ such that (S1.12) holds.

It is therefore clear that one can pick γ so small that solutions of the combined system (S1.3), (S1.16) will eventually converge into the domain specified by (S1.14). In this domain the function λ_{post} defined in (S1.16) will satisfy (S1.13), albeit possibly with different constants γ, Γ. Hence, Proposition 1 applies, and thus one can pick $\gamma > 0$ sufficiently small so that $\lim_{t\to\infty,\ i\to\infty} \sigma(\varphi, t, i) = \Phi_c$. This, in turn implies that $\varphi(t) \to \Phi_c$ at $t \to \infty$.

Local non-uniform small gain theorem

Consider a system with input, u, and let the evolution of its state, x, be governed by

$$\dot{x} = f(x, u(t), t), \tag{S1.17}$$

where the function $f : \mathbb{R}^n \times \mathbb{R} \times \mathbb{R} \to \mathbb{R}^n$ is continuous in x, u, t and bounded in t, and $u : \mathbb{R} \to \mathbb{R}$ is a continuous function. For the sake of notational compactness we denote $\|z(\tau)\|_{\infty,[a,b]} = \max_{\tau\in[a,b]} \|z(\tau)\|$, where z is a function defined on $[a, b]$. Let $\Omega(t_0, t) = \Omega_x \times \Omega_u(t_0, t)$, $\Omega_x : \{x \in \mathbb{R} |\ \|x\| \leq \Delta_x\}$, $\Delta_x \in \mathbb{R}_{>0}$, $\Omega_u(t_0, t) = \{u|\ u : \mathbb{R} \to \mathbb{R},\ u \in \mathcal{C}^0,\ \|u\|_{\infty,[t_0,t]} \leq \Delta_u\}$, and suppose that for any $t_0 \in \mathbb{R}$, $t > t_0$ and all $(x_0, u) \in \Omega(t_0, t)$, the solutions of (S1.17) satisfying $x(t_0) = x_0$ are defined, and the following holds

$$\|x(t_0 + T)\| \leq \beta(T)\|x(t_0)\| + c\|u(t)\|_{\infty,[t_0,t_0+T]},\ T \in [0, t - t_0],\ c \in \mathbb{R}_{\geq 0},$$

where $\beta(T)$ is a strictly monotone continuous function: $\lim_{t\to\infty} \beta(t) = 0$, $\beta(0) \geq 1$. Let us suppose that for all t_0 input u in (S1.17) is evolving according to

$$u(t_0) \geq u(t) \geq u(t_0) - (t - t_0)\gamma\|x(t')\|,\ t' \in [t_0, t],\ t \geq t_0,\ \gamma \in \mathbb{R}_{>0}. \tag{S1.18}$$

Then the following statement holds for interconnection (S1.17), (S1.18) (cf. [3], [4])

Proposition 1 *Consider interconnection (S1.17), (S1.18), and suppose that the domain*

$$\Omega_\gamma :\ 0 \leq \gamma \leq \beta^{-1}\left(\frac{d}{\kappa}\right)^{-1} \frac{\kappa - 1}{\kappa} \frac{u(t_0)}{\beta(0)(\|x(t_0)\| + c|u(t_0)|(1 + \kappa/(1 - d))) + c|u(t_0)|} \tag{S1.19}$$

is not empty for some $d < 1$, $\kappa > 1$. Let $\Omega_\Delta = \{(x, u)|\ \beta(0)\|x\| + c|u| \leq \Delta_x,\ |u| \leq \Delta_u\}$.

Then for all $(x_0, u(t_0)) \in \Omega_\gamma \cap \Omega_\Delta$ the state $(x(t), u(t))$ of the interconnection is bounded. Furthermore, if there is a function $w : \mathbb{R}^n \to \mathbb{R}_{\geq 0}$ such that

$$u(t) \leq u(t_0) - (t - t_0)w(x(t')),\ t' \in [t_0, t],\ t \geq t_0, \tag{S1.20}$$

then for every divergent and ordered sequence $\{t_i\}$, $i = 0, 1, \ldots,$ $t_i < t_{i+1}$, the following holds:

$$\exists\ \{t'_i\},\ t'_i \in [t_i, t_{i+1}]\ :\ \lim_{t_i\to\infty} w(x(t'_i)) = 0. \tag{S1.21}$$

Proof of Proposition 1. Let us introduce a strictly decreasing sequence $\{\sigma_i\}$, $i = 0, 1, \ldots,$ such that $\sigma_0 = 1$, and σ_i asymptotically converge to zero. Let $\{t_i\}$, t_i, $i = 1, \ldots$ be an ordered infinite sequence of time instances such that

$$u(t_i) = \sigma_i u(t_0).$$

In case this equality does not hold, nothing remains to be proven since $u(t)$ will always be bounded and separated away from zero for $t \geq t_0$.

We wish to show that the amount of time needed to reach the set $\{(x,u)|\ u=0\}$ from the given initial condition is larger than any positive number, i.e. infinite.

Consider time differences $T_i = t_i - t_{i-1}$. It is clear that:

$$T_i \|x(\tau)\|_{\infty,[t_{i-1},t_i]} \geq \frac{u(t_0)(\sigma_{i-1}-\sigma_i)}{\gamma}. \tag{S1.22}$$

In addition to $\{t_i\}$ we introduce another auxiliary sequence $\{\tau_i\}$, $\tau_i = \tau^*$, $\tau^* \in \mathbb{R}_{>0}$, $i = 1, \ldots$. Given that the partial sums $\sum_i \tau_i = \sum_i \tau^*$ diverge we can conclude that proving the implication

$$T_i \geq \tau^* \Rightarrow T_{i+1} \geq \tau^* \ \forall\ i \tag{S1.23}$$

will automatically assure that $x(t)$, $u(t)$ are bounded for all $t \geq t_0$. We prove (S1.23) by induction wrt i.

Let $x(t_0)$, $u(t_0)$ be such that $\beta(0)\|x(t_0)\| + c|u(t_0)| < \Delta_x$, $0 < u(t_0) < \Delta_0$. The function $u(t)$ is non-increasing over $[t_0, t_i]$. This ensures that $u \in \Omega_u(t_0, t_i)$, and hence $x(t_i), u(t_i) \in \Omega_\Delta$ for all t_i. Suppose that $T_j \geq \tau^*$ for $1 \leq j \leq i-1$. Therefore

$$\begin{aligned}\|x(\tau)\|_{\infty,[t_{i-t},t_i]} &\leq \beta(0)\,\|x(t_{i-1})\| + cu(t_0)\sigma_{i-1} \leq \beta(0)\,[\beta(T_{i-1})\,\|x(t_{i-2})\| + cu(t_0)\sigma_{i-2}] + cu(t_0)\sigma_{i-1}\\ &\leq \beta(0)\beta^2(\tau^*)\,\|x(t_{i-3})\| + P_2,\end{aligned}$$

where

$$P_2 = \beta(0)\,[\beta(\tau^*)c\sigma_{i-3} + c\sigma_{i-2}]\,u(t_0) + c\sigma_{i-1}u(t_0)$$

Repeating this iteration with respect to i leads to

$$\begin{aligned}&\|x(\tau)\|_{\infty,[t_{i-t},t_i]} \leq \beta(0)\beta^3(\tau^*)\,\|x(t_{i-4})\| + P_3\\ &P_3 = cu(t_0)\beta(0)\left[\beta^2(\tau^*)\sigma_{i-4} + \beta(\tau^*)\sigma_{i-3} + \sigma_{i-2}\right] + \sigma_{i-1}cu(t_0)\\ &\quad = cu(t_0)\beta(0)\left[\sum_{j=0}^{2}\beta^j(\tau^*)\sigma_{i-j-2}\right] + cu(t_0)\sigma_{i-1},\end{aligned}$$

and after $i-1$ steps we obtain

$$\begin{aligned}&\|x(\tau)\|_{\infty,[t_{i-t},t_i]} \leq \beta(0)\beta(\tau^*)^{i-1}\,\|x(t_0)\| + P_{i-1}\\ &P_{i-1} = cu(t_0)\beta(0)\left[\sum_{j=0}^{i-2}\beta^j(\tau^*)\sigma_{i-j-2}\right] + cu(t_0)\sigma_{i-1}.\end{aligned} \tag{S1.24}$$

Rearranging terms in (S1.22) results in

$$T_i \geq \frac{\sigma_{i-1}-\sigma_i}{\sigma_{i-1}}\frac{u(t_0)}{\gamma}\frac{1}{\sigma_{i-1}^{-1}\,\|x(\tau)\|_{\infty,[t_{i-1},t_i]}}.$$

Hence, if we can show that there exist an x_0 such that such that for some $\Delta_0 \in \mathbb{R}_{\geq 0}$:

$$\frac{\sigma_{i-1}-\sigma_i}{\sigma_{i-1}}\frac{u(t_0)}{\tau^*} \geq \Delta_0 \tag{S1.25}$$

the following holds

$$\gamma\sigma_{i-1}^{-1}\,\|x(\tau)\|_{\infty,[t_{i-1},t_i]} \leq \gamma B(x_0) \leq \Delta_0\ \forall\ i,$$

where $B(\cdot)$ is a function of x_0, then implication (S1.23) will obviously follow. Consider $\sigma_{i-1}^{-1}\|x(\tau)\|_{\infty,[t_{i-1},t_i]}$, and let

$$\sigma_i = \frac{1}{\kappa^i},\ \kappa > 1$$

According to (S1.24) we have:

$$\sigma_{i-1}^{-1}\|x(\tau)\|_{\infty,[t_{i-1},t_i]} \leq \beta(0)\left[\sigma_{i-1}^{-1}\beta(\tau^*)^{i-1}\right]\|x(t_0)\| + \sigma_{i-1}^{-1}P_{i-1} = \beta(0)(\kappa\beta(\tau^*))^{i-1}\|x(t_0)\| +$$

$$\kappa^{i-1}P_{i-1} = \beta(0)(\kappa\beta(\tau^*))^{i-1}\|x(t_0)\| + cu(t_0)\beta(0)\kappa\left[\sum_{j=0}^{i-2}\beta^j(\tau^*)\kappa^j\right] + cu(t_0).$$

Hence choosing the value of τ^* as

$$\kappa\beta(\tau^*) \leq d,\ d \in (0,1) \tag{S1.26}$$

results in the following estimate:

$$\sigma_{i-1}^{-1}\|x(\tau)\|_{\infty,[t_{i-1},t_i]} \leq \beta(0)\|x(t_0)\| + cu(t_0)\left(1 + \frac{\beta(0)\kappa}{1-d}\right) = B(x_0).$$

Solving (S1.26), (S1.25) with respect to Δ_0 results in

$$\Delta_0 = \frac{\kappa-1}{\kappa}\left[\beta^{-1}\left(\frac{d}{\kappa}\right)\right]^{-1} u(t_0).$$

This in turn implies that for all $(x(t_0), u(t_0)) \in \Omega_\Delta$ which, in addition, satisfy:

$$\gamma \leq \frac{\kappa-1}{\kappa}\left[\beta^{-1}\left(\frac{d}{\kappa}\right)\right]^{-1}\frac{u(t_0)}{\beta(0)\|x(t_0)\| + c|u(t_0)|\left(1 + \frac{\kappa\beta(0)}{1-d}\right)}$$

the following implication must hold: $T_i \geq \tau^* \Rightarrow T_{i+1} \geq \tau^*$. Therefore, trajectories $x(t)$, $u(t)$ passing through $x(t_0)$, $u(t_0)$ at $t = t_0$ are bounded in forward time.

Finally, let us show that (S1.20) implies (S1.21). Suppose that this is not the case, and for an ordered diverging sequence $\{t_i\}$ there is a $\delta > 0$ such that $w(x(t')) > \delta$, $\forall\ t' \in [t_{i-1}, t_i]$, $i = 1, 2, \ldots$. Hence

$$u(t_i) \leq u(t_{i-1}) - (t_i - t_{i-1})\delta \leq u(t_0) - (t_0 - t_{i-1})\delta. \tag{S1.27}$$

According to the first part of the proposition $u(t_i)$ is bounded for all i. On the other hand, using (S1.27) one can conclude that for any given arbitrarily large M, there is an $n > 0$ such that $u(t_n) \leq -M$. Thus we have reached contradiction which proves (S1.21) □.

References

1. Hoppensteadt F, Izhikevich E (1997) Weakly connected neural networks. Springer-Verlag.

2. Pikovsky A, Rosenblum M, Kurths J (2001) Synchronization: a unified concept in nonlinear sciences. Cambridge University Press.

3. Tyukin I (2011) Adaptation in dynamical systems. Cambridge University Press.

4. Tyukin I, Steur E, Neijmeijer H, van Leeuwen C (2008) Small-gain theorems for systems with unstable invariant sets. SIAM Journal on Control and Optimization 47: 849–882.
